# Operative treatment of severe scoliosis and pelvic obliquity in patients with spinal muscular atrophy: assessment of outcomes and complications

**DOI:** 10.1186/s13023-025-03682-8

**Published:** 2025-04-11

**Authors:** Heng Sun, Yizhen Huang, Yulei Dong, Zhen Wang, Junduo Zhao, Xuan Huang, Weiyun Chen, Jianxiong Shen

**Affiliations:** 1 Department of Orthopedic Surgery, Peking Union Medical College, Peking Union Medical College Hospital, Chinese Academy of Medical Science, Beijing, People’s Republic of China; 2 Department of Anesthesiology, State Key Laboratory of Complex Severe and Rare Diseases, Peking Union Medical College Hospital, Peking Union Medical College and Chinese Academy of Medical Sciences, Beijing, People’s Republic of China

**Keywords:** Clinical outcome, Pelvic fixation, Posterior spinal fixation, Scoliosis, Spinal muscular atrophy

## Abstract

**Background:**

Few reports exist that focus on patients with spinal muscular atrophy (SMA) and severe spinal deformity. In this study, we aimed to report surgical outcomes and complications for SMA patients with severe scoliosis and pelvic obliquity.

**Methods:**

A retrospective review of data on operatively treated SMA patients with severe scoliosis and pelvic obliquity (minimum major coronal curve Cobb angle > 100° and pelvic obliquity > 20°) was performed. Radiography findings, pulmonary function, motor status, the sitting function score, and perioperative and postoperative complications were the main clinical outcomes examined. Muscular dystrophy spine questionnaire (MDSQ) responses and caregiver responses to four anchor questions (quality of life/comfort/ease of care/overall health) using Likert scales were recorded.

**Results:**

Of 28 consecutive patients, 22 (79%) completed the minimum 2-year follow-up (mean age at surgery = 16.1, 68% female). The mean follow-up duration was 40.3-mo. All patients underwent one-stage posterior spinal fusion (PSF) with pelvic fixation. Radiographic measurements (main coronal curve, kyphosis, pelvic obliquity) were significantly corrected (all *p* < 0.001) and were maintained at the last follow-up. The mean forced vital capacity (FVC) remained stable during follow-up, with 50% of patients showing improvement. The percentage of patients who could sit independently increased significantly from 22.7% preoperatively to 77.3% postoperatively (*p* < 0.001). The total sitting-related MDSQ score significantly increased from 8.5 to 12.5 at 6 months postoperatively, and to 15.0 at the last follow-up (*p* < 0.001). Six instances of complications (two instances each of pneumonia, epiglottic edema, and delayed wound healing) occurred perioperatively in six patients (27.3%), but no surgical intervention was required.

**Conclusion:**

Operative treatment significantly improved radiographic parameters and sitting function and maintained pulmonary function without serious complications in SMA patients with severe scoliosis and pelvic obliquity.

## Background

Spinal muscular atrophy (SMA) is a hereditary neuromuscular disease primarily associated with progressive proximal muscle weakness and atrophy [1]. Progressive scoliosis occurs in nearly 90% of patients with type II or III SMA [2]. Early pharmacological treatment may slow the progression of spinal deformity in patients with type II SMA; however, evidence supporting its benefit in patients with severe spinal deformity is insufficient [3]. Growth-friendly devices are recommended for managing early-onset SMA scoliosis to delay progression to severe scoliosis [4, 5]. In developing countries, some patients with SMA experience delayed treatment owing to the costs related to pharmacological treatment and growth-friendly devices.

Delayed treatment of patients with type II or III SMA often results in the loss of walking ability, leading to severe scoliosis and pelvic obliquity (PO), resulting in unbalanced sitting. Severe spinal deformity and related muscle weakness result in pulmonary and digestive dysfunction. In a previous study, posterior spinal fusion (PSF) was recommended to halt scoliosis progression and minimize restrictive lung disease in SMA patients who initially presented at an age ≥ 10 year and who required immediate spinal fusion[6], however, few studies have focused on the subset of SMA patients with severe scoliosis and PO [5, 7–9]. Therefore, our objective was to assess surgical outcomes and complications in the subset of SMA patients with severe scoliosis and PO.

## Methods

### Study population

**Fig. 1:**
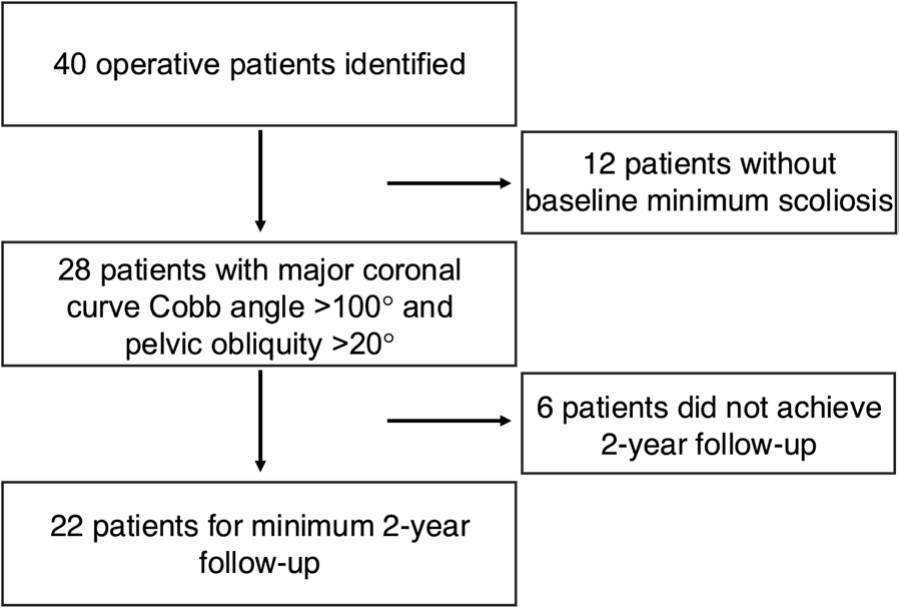
Flow chart of study patient selection

The Institutional Board of Peking union medical college hospital approved this study (IRB: S-K1863), and all patients provided consent for study participation. Patients were enrolled between November 2014 and March 2022; the included patients had a genetic and clinical diagnosis of type II or III SMA and had undergone dorsal spinal fusion. Other inclusion criteria comprised patients: (i) with non-ambulatory status; (ii) with severe spinal deformity (preoperative major coronal curve Cobb angle > 100° and PO > 20°); (iii) aged > 10 years at the time of surgery; and (iv) who were followed-up for more than 2 years. A total of 22 patients were enrolled based on the abovementioned inclusion criteria (Fig. 1). No previous reports on this cohort have been published. Patient characteristics are shown in Table 1.

**Table 1:** Demographic data, radiographic and pulmonary function measurements

	Preoperatively (n = 22)	6-months postoperatively (n = 22)	Last follow-up (n = 22)	*p*-value
Age at scoliosis onset, years, (range)	7.0 (2–15)			
Age at surgery, years, (range)	16.1 (11–26)	–	–	–
Sex (male/female)	7/15			–
Weight, kg, (SD)	41.4 (12.3)			
BMI < 18.5, n (%)	12 (54.5)			
BMI ≥ 18.5, n (%)	10 (45.5)			
Type II/III	14/8			–
Hip status, n (%)				
Normal	5 (22.7)	–	–	-
Subluxated	10 (45.5)	–	–	–
Dislocated	7 (31.8)	–	–	–
Motor status, n (%)				
Independent sit	4 (22.7)	18 (77.3)	18 (77.3)	< 0.001
Sit with support	18 (77.3)	4 (22.7)	4 (22.7)	< 0.001
Major coronal curve, °, (SD)	116.7 (16.4)	59.6 (19.8)	59.8 (19.6)	< 0.001
Kyphosis, °, (SD)	98.9 (33.5)	45.4 (19.8)	44.8 (19.5)	< 0.001
Pelvic obliquity, °, (SD)	35.1 (10.4)	13.8 (9.9)	12.6 (9.0)	< 0.001
T1–T12 height, cm, (SD)	17.1 (2.9)	22.4 (2.9)	22.5 (3.4)	< 0.001
Pulmonary function test				
FVC, mL, (SD)	1195 (644)	1155 (610)	1154 (649)	0.971
FVC percentage predicted, %, (SD)	39.4 (18.8)	38.1 (18.7)	36.9 (20.8)	0.912

BMI, body mass index; FVC, forced vital capacity; SD, standard deviation

*p*-values are derived from a one-way analysis of variance test for continuous variables and a Friedman test for categorical variables

### Clinical outcome measures

Radiography findings, pulmonary function, motor status, the sitting function score, and perioperative and postoperative complications were the main clinical outcomes examined. Radiographic imaging and pulmonary function tests were performed preoperatively, at 6 months postoperatively, and at the last follow-up. Radiographic measurements were assessed on sitting whole-spine radiographs and included the main coronal curve Cobb angle, kyphosis, PO (defined as the angle between the horizontal line and the interiliac crest line), and thoracic height (T1–T12). Forced vital capacity (FVC) and FVC percentage predicted (FVC%) data were also evaluated.

The sitting function score was measured with sitting-related questions taken from the muscular dystrophy spine questionnaire (MDSQ), a validated instrument specifically designed to assess motor function in patients with neuromuscular scoliosis [10]. The questions were in relation to the following nine items: Q7, sitting up in bed; Q15, sitting comfortably in a good position in a wheelchair all day; Q16, shifting weight or changing the hip position in a wheelchair; Q22, sitting in a chair all day without breaks; Q24, sitting at the table for meals; Q26, maintaining balance while sitting in a wheelchair; Q27, appearing appropriately positioned in a wheelchair; Q28, hip and back pain; and Q29, feeling out of breath when sitting in an imbalanced position. Questions 7, 15, 16, 22, 24, 26, and 27 were scored separately on a 5-point Likert scale ranging from 0 (cannot manage it) to 4 (not difficult). Questions 28 and 29 were scaled from 0 (extremely bad) to 4 (not a problem) [15]. Responses to sitting-related questions and motor status data were collected from all patients preoperatively, at 6 months postoperatively, and at the last follow-up. Additionally, caregivers were requested to rate the effects of surgery on four domains using a 5-point Likert scale (anchor questions). The questions were formulated as follows: “How has your child’s quality of life/comfort/ease of care/overall health changed postoperatively?” The responses ranged from 1 (deteriorated considerably) to 5 (improved considerably), and data were recorded at 6 months postoperatively and at the last follow-up.

Perioperative and postoperative complication data were collected and categorized into gastrointestinal, respiratory, neurological, wound, and mechanical complications.

### Statistical analysis

Data were analyzed using SPSS Windows (version 27.0) software and are presented as medians with interquartile ranges (IQRs) or means with standard deviation (SD). Sitting-related question scores in the MDSQ preoperatively and postoperatively were analyzed using a Wilcoxon signed-rank test. Longitudinal categorical variables were analyzed using a Friedman test. One-way analysis of variance (ANOVA) was used to analyze radiographic and pulmonary function data. The significance level was set at *p* < 0.05.

## Results

The study cohort comprised 22 consecutive patients (15 females, 7 males). The mean age at scoliosis onset was 7.0 (range, 2–15) years. The mean age at the time of surgery was 16.1 (range, 11–26) years, and 14 (63.6%) patients were diagnosed with type II SMA (Table 1). The mean follow-up duration was 40.3 (range, 24–72) months. Preoperatively, 12 (54.4%) patients had a low weight, with a body mass index < 18.5 kg/m^2^. Moreover, 10 (45.5%) and 7 (31.8%) patients presented with hip subluxation and hip dislocation, respectively. All patients had undergone one-stage posterior spine fusion to the pelvis. S2 Alar-Iliac screws were used in 17 (77.3%) patients and iliac screws were used in 5 (22.7%) patients. The upper instrumented vertebrae were as follows: T1 (1 patient, 4.5%); T2 (5 patients, 22.7%); T3 (12 patients, 54.5%); T4 (3 patients, 13.6%); and T5 (1 patient, 4.5%). In total, 18 (81.8%) patients received multi-rod constructs. All patients underwent unilateral interlaminar fenestration on the convex side during spinal fusion surgery for postoperative intrathecal nusinersen injection.

### Radiological outcomes

The mean major coronal curve was significantly corrected after surgery (preoperative, 116.7° ± 16.4° vs. 6-month, 59.6° ± 19.8° vs. final follow-up, 59.8° ± 19.65°, *p* < 0.001). Kyphosis significantly improved from 98.9° ± 33.5° preoperatively to 45.4° ± 19.7° at 6 months postoperatively and was maintained at 44.8° ± 19.5° at the last follow-up (*p* < 0.001). Similar improvements were observed in postoperative PO and T1–T12 height (*p* < 0.001; Table 1).

### Outcomes of pulmonary function test

The average preoperative FVC and FVC% were 1195 mL ± 644 mL and 39.4 ± 18.8%, respectively. The mean FVC (one-way ANOVA test, *p* = 0.971) and FVC% (one-way ANOVA test, *p* = 0.912) were stable during the follow-up period. Compared with the preoperative FVC, 50% of patients showed improvement in FVC postoperatively, whereas the remaining patients showed worsening FVC at the last follow-up (Table 2).

**Table 2:** Pulmonary function and motor status in patients with spinal muscular atrophy and severe scoliosis before and after scoliosis surgery

Case no	Sex	SMA type	Age of scoliosis onset (years)	Age of scoliosis surgery (years)	Follow-up duration (months)	Preoperative FVC(mL)	Postoperative FVC (mL, 6-months)	Postoperative FVC (mL, last follow-up)	Changes in FVC (last follow-up)	Preoperative motor status	Postoperative motor status (6-months)	Postoperative motor status (last follow-up)	Changes in motor status (last follow-up)
1	F	2	7	13	36	1210	1430	1400	Improved	Sit with support	Sit with support	Sit with support	Static
2	F	3	4	17	72	630	720	650	Improved	Independent sit	Independent sit	Independent sit	Static
3	F	2	7	17	43	520	480	510	Worsened	Sit with support	Independent sit	Independent sit	Improved
4	F	2	6	20	31	810	760	640	Worsened	Sit with support	Independent sit	Independent sit	Improved
5	M	2	9	13	30	2620	2480	2740	Improved	Sit with support	Independent sit	Independent sit	Improved
6	F	2	9	23	27	540	630	610	Improved	Sit with support	Independent sit	Independent sit	Improved
7	M	2	10	16	31	1260	1150	1060	Worsened	Sit with support	Sit with support	Sit with support	Static
8	F	2	4	12	43	1090	1140	1210	Improved	Independent sit	Independent sit	Independent sit	Static
9	F	2	8	11	38	640	530	570	Worsened	Sit with support	Independent sit	Independent sit	Improved
10	F	3	2	13	44	890	950	920	Improved	Sit with support	Independent sit	Independent sit	Improved
11	M	3	3	18	36	1250	1180	1040	Worsened	Sit with support	Sit with support	Sit with support	Improved
12	F	3	7	13	27	800	630	580	Worsened	Sit with support	Independent sit	Independent sit	Improved
13	F	3	6	20	31	1560	1320	1410	Worsened	Sit with support	Independent sit	Independent sit	Improved
14	F	3	9	13	24	1780	1840	1890	Improved	Sit with support	Sit with support	Sit with support	Static
15	M	2	5	12	42	880	960	980	Improved	Sit with support	Independent sit	Independent sit	Improved
16	F	2	9	17	42	820	780	850	Improved	Sit with support	Independent sit	Independent sit	Improved
17	F	2	6	11	32	720	860	970	Improved	Sit with support	Independent sit	Independent sit	Improved
18	M	3	15	22	56	2920	2780	2720	Worsened	Independent sit	Independent sit	Independent sit	Static
19	F	2	7	15	31	1510	1360	1630	Improved	Sit with support	Independent sit	Independent sit	Improved
20	M	2	8	15	66	880	740	550	Worsened	Sit with support	Independent sit	Independent sit	Improved
21	M	2	9	26	48	1960	1850	1720	Worsened	Sit with support	Independent sit	Independent sit	Improved
22	F	3	4	17	56	990	840	740	Worsened	Independent sit	Independent sit	Independent sit	Static

F, female; FVC, forced vital capacity; M, male; SMA, spinal muscular atrophy

### Outcomes of quality-of-life

**Fig. 2:**
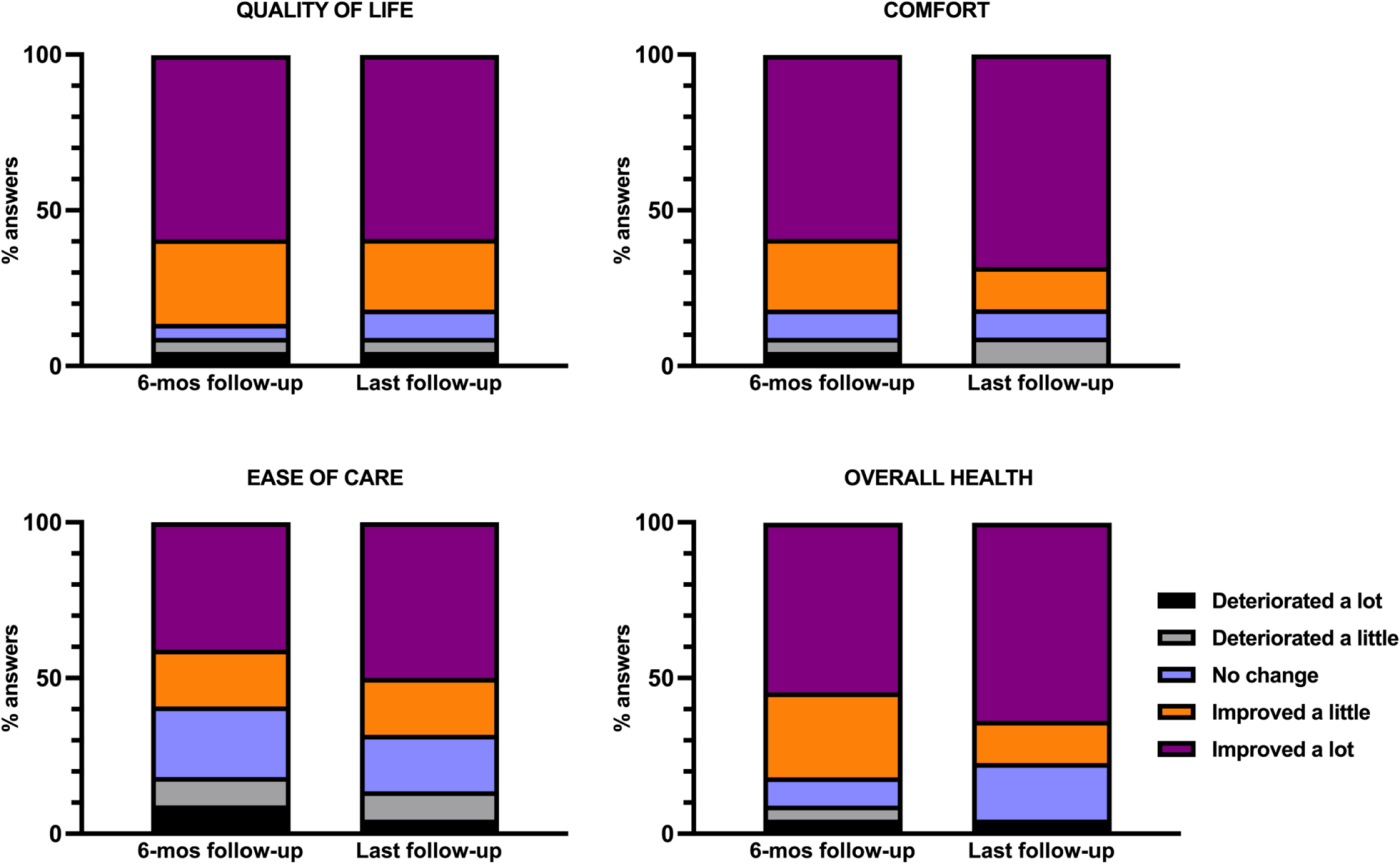
Distribution of the four anchor question answers at 6 months and the last postoperative follow-up. No significant difference was observed across the two time points analyzed

The percentage of patients capable of sitting independently increased from 22.7 (4/22) preoperatively to 77.3% (18/22) (Friedman test, *p* < 0.001) postoperatively (Table 1). Compared with the preoperative motor status, 68.2% (15/22) of patients showed improvement in motor function, and no patient showed worsening at the last follow-up (Table 2). Analysis of the MDSQ sitting-related question scores indicated that spinal surgery had a significant effect on sitting function (Table 3). The scores for sitting-related questions in the MDSQ, except for Q**2**8 (pain in my hips and back), improved at 6 months postoperatively (Wilcoxon signed rank test, all *p* < 0.05). The total and individual scores for sitting-related questions in the MDSQ significantly increased at the last follow-up (all *p* < 0.05) (Table 3). At 6 months postoperatively, 86.4% (19/22) of caregivers reported an improvement in their child’s quality of life, whereas only 4.5% (1/22) reported deterioration (Fig. 2). Comfort improved in 77.3% (17/22) and deteriorated in 9.1% (2/22) of patients; ease of care improved in 72.7% (16/22) and deteriorated in 13.6% (3/22) of patients; and overall health improved in 81.8% (18/22) and deteriorated in 4.5% (1/22) of patients (Fig. 1). Compared with the response at 6 months postoperatively, the results did not differ significantly at the last follow-up evaluation (Wilcoxon signed rank test, all *p* > 0.05).

**Table 3:** Sitting-related question score in muscular dystrophy spine questionnaire

	Question	Preoperatively(n = 22)	6-months postoperatively (n = 22)	*p*-value (pre-vs. 6-months postoperatively)	Last follow-up (n = 22)	*p*-value (preoperatively vs. last follow-up)
7	Able to sit up in bed	1.0 (0.0–2.0)	1.5 (1.0–2.0)	0.021	1.5 (1.0–2.0)	0.002
15	Sitting comfortably in a good position in my wheelchair throughout the day	0.0 (0.0–1.0)	1.0 (0.8–1.0)	0.002	1.0 (0.8–1.3)	0.003
16	Shifting weight or changing my hip position while seated in my wheelchair	0.5 (0.0–1.0)	1.0 (0.0–1.0)	0.025	1.0 (0.0–2.0)	0.005
22	Sitting in my wheelchair all day without breaks	0.0 (0.0–1.0)	1.0 (0.0–1.0)	0.007	1.0 (0.0–1.0)	0.005
24	Sitting at the table for meals	1.0 (0.0–1.0)	1.0 (1.0–2.0)	< 0.001	1.0 (1.0–2.0)	0.007
26	Maintaining my balance while sitting in my wheelchair	1.0 (0.8–2.0)	2.0 (1.0–2.0)	0.020	2.0 (1.0–2.0)	0.013
27	Sitting in an appropriate position while in my wheelchair	1.0 (0.0–1.0)	1.0 (0.0–1.3)	0.025	1.0 (0.0–2.0)	0.003
28	Pain in my hips and back	2.0 (1.0–2.0)	2.0 (2.0–2.0)	0.083	2.0 (2.0–3.0)	0.006
29	Feeling out of breath when sitting in an imbalanced position	2.0 (1.8–2.0)	2.0 (2.0–3.0)	0.033	3.0 (2.0–3.0)	0.003
-	Total score	8.5 (6.0–12.3)	12.5 (7.8–15.0)	< 0.001	15.0 (9.5–16.3)	< 0.001

Data are presented as medians with interquartile range

*p*-values are derived from a Wilcoxon signed rank test

### Outcomes of complications

Six (27.3%) patients experienced perioperative complications. No complications occurred between the perioperative period and the last follow-up (Table 4). Patients with perioperative complications, including pneumonia (2 patients), epiglottic edema (2 patients), and delayed wound healing (2 patients), had recovered by 2 weeks postoperatively. Moreover, no patient required surgical intervention as a result of complications (Table 4).

**Table 4:** Overall complication rate at perioperatively, last follow-up evaluations

	Perioperative (N = 22)	Last follow-up (N = 22)
Patients with complication (*no. [%]*)	6 (27.3)	0
Complications		
Gastrointestinal	0	0
Respiratory	4 (18.2)	0
Epiglottic edema	2 (9.1)	0
Pulmonary infection	2 (9.1)	0
Pulmonary failure	0	0
Neurological	0	0
Wound	2 (9.1)	0
Delayed healing	2 (9.1)	0
Surgery site infection	0	0
Mechanical	0	0
Patients required reoperation	0	0

## Discussion

SMA patients have a high risk of developing severe spinal deformities, for which conventional interventions, such as bracing and drug treatment, often prove ineffective [11]. Surgical treatment is the standard approach for SMA scoliosis [1]. Growth-friendly devices have been shown to improve radiographic outcomes in patients with early-stage SMA [5, 12]. PSF is recommended in SMA patients with scoliosis who initially present at an age ≥ 10 year. However, few reports exist that focus on the subset of SMA patients with severe scoliosis and pelvic fixation. Therefore, in this study, we sought to determine the benefits and risks of operative treatment in SMA patients with severe spinal deformity, using a minimum of 2 years of postoperative medical data.

**Fig. 3:**
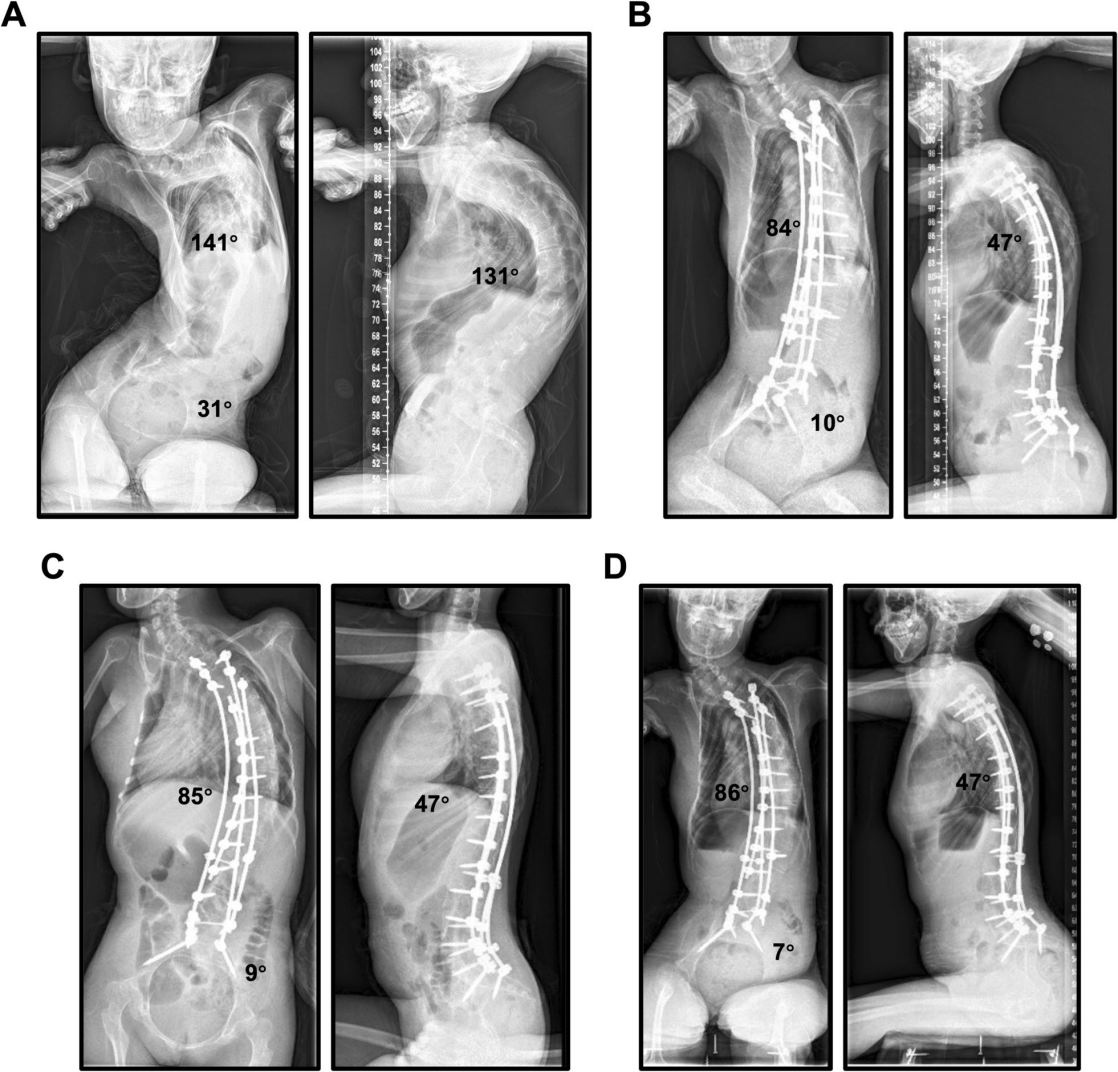
A 11-year-old female diagnosed with SMA underwent posterior spinal fusion and pelvic fixation by using traditional iliac screws (left side: 7.0 * 65 mm; right side: 7.0 * 40 mm). (**A**) Preoperative posteroanterior and lateral radiographs of the sitting whole-spine showing severe scoliosis with associated increased kyphosis and pelvic obliquity. (**B**) This image shows that spinal deformity and pelvic obliquity were corrected at 6-mo postoperatively. Images (**C**) and (**D**) show that the corrected spinal deformity and pelvic obliquity were maintained at 18-months and 64-months postoperatively, respectively

**Fig. 4:**
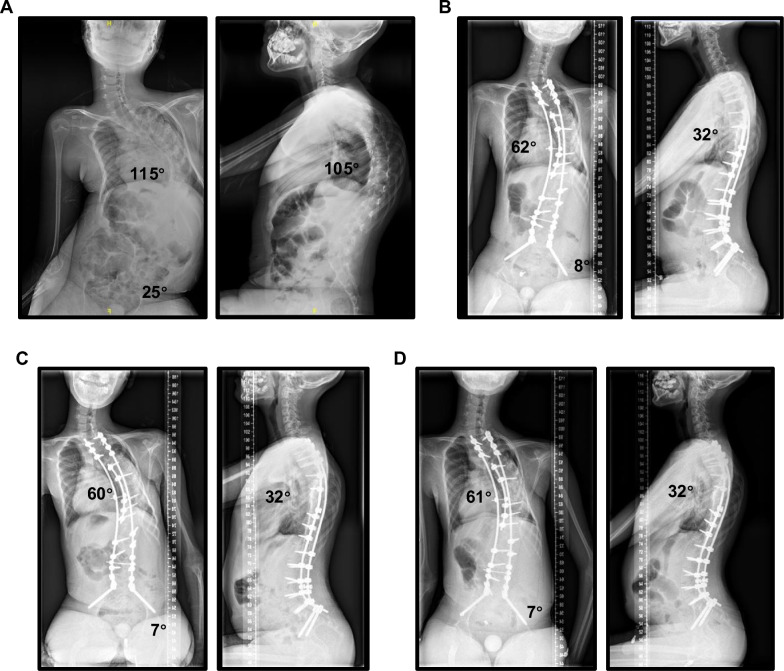
A 12-year-old male diagnosed with SMA underwent posterior spinal fusion and pelvic fixation by using S2-AI screws (both side: 8.0 * 80 mm). (**A**) Preoperative posteroanterior and lateral radiographs of the sitting whole-spine showing severe scoliosis (main curve cobb angle: 115°) with pelvic obliquity (25°). (**B**) shows that spinal deformity and pelvic obliquity were corrected at 6-mo postoperatively. (**C**) and (**D**) show that the corrected spinal deformity and pelvic obliquity were maintained at 18-months and 42-months postoperatively, respectively

Considering the severity of the deformity and age at the time of surgery, all patients had undergone PSF with pelvic fixation (Fig. 3). Multi-rod constructs, which have been reported to increase the stability of internal fixation systems, were used in 81.8% of patients [13]. We found that the multi-rod constructs enabled surgeons to connect the rods to the screws more effectively, achieve good apex correction, and balance the spine. S2-AI or iliac screws were used for pelvic fixation via the S2-AI pathway. The PO improved from 35.1° ± 10.4 preoperatively to 13.8° ± 9.9 at 6 months postoperatively. Compared with previous techniques, pelvic fixation with S2-AI screws reduces the operative time, blood loss, possibility of surgical site infections, and implant prominence while maintaining biomechanical stability [14, 15]. However, S2-AI screw application is challenging in some patients with severe SMA. Iliac deformity results in an insufficient length and narrow diameter of the S2-AI trajectory. Therefore, we recommend using computed tomography scanning to evaluate the length and narrow diameter of the iliac space in patients with SMA preoperatively and we encourage the design of smaller S2-AI screws. (Fig. 4)

Severe scoliosis and PO in SMA patients can cause sitting pain and difficulty, which represent the greatest challenges in terms of quality of life [16]. The measurement of sitting function in SMA patients with severe spinal deformity is challenging. A previous study assessed motor function after surgery for scoliosis in patients with SMA based on a neuromuscular postoperative questionnaire [17]. However, this questionnaire was designed without direct patient input, primarily targeting parents, and did not assess sitting function. In contrast, the MDSQ is a patient-administered questionnaire tailored to assess critical functional abilities pertinent to children with muscular dystrophy and scoliosis [18]. The nine sitting-related questions address various aspects of sitting, including motion, comfort, esthetics, pain, and breathing. Suk et al. [19] retrospectively compared 40 surgically treated patients with Duchenne muscular dystrophy and 26 conservatively managed patients, revealing a notable increase in sitting-related MDSQ scores and improved sitting function in the surgical cohort after at least 2 years of follow-up. Our study corroborates these observations, showing significant improvements in sitting-related MDSQ scores and motor status at both 6 months and 2 years postoperatively (Tables 3 and 4). Although the median score for Q28 (pain in the hips and back) showed significant improvement at the final follow-up, persistent pain in some patients at the 6-month follow-up may be indicative of residual biomechanical stresses, compensatory muscle fatigue, or the progression of hip pathology. This is the first study to longitudinally assess long-term sitting functional outcomes in SMA patients with severe spinal deformity after operative treatment using validated and reliable assessment tools.

Impaired pulmonary function is associated with scoliosis progression in SMA patients. Robinson et al. reported that for every 10° increase in the Cobb angle, there was a 4.7% decrease in predicted vital capacity [20]. Therefore, limited progression of scoliosis is recommended to maintain sufficient pulmonary function and improve quality of life. However, there is insufficient evidence to support surgery for scoliosis to improve pulmonary function. Chong et al. reported that pulmonary function decreased at 1-year postoperatively following scoliosis surgery in 11 patients with SMA [21]. Chou et al.[22] reported that the pulmonary function of 10 patients with type 2 SMA was maintained after surgery for scoliosis during a 10-year follow-up period. In this study, compared with the preoperative FVC, an equal number (11/22) of patients showed improvement and worsening, with the mean FVC remaining stable for at least 2 years of follow-up. In this study, the variations in FVC trends among patients underscore the challenge of distinguishing surgical outcomes from the natural progression of SMA. The stabilization of FVC in this cohort represents a favorable outcome, especially when compared to the historical decline in FVC observed in untreated SMA patients with severe scoliosis. With in the MDSQ, Question 29 (i.e., feeling out of breath when sitting in an imbalanced position) relates to a subjective perception of breathing and the responses indicated that the patients had experienced substantial improvements. The findings indicate favorable outcomes following surgery for scoliosis considering the natural disease course, which involves progressive deterioration of pulmonary function over time.

Perioperative and postoperative complications of surgery for SMA patients with severe spinal deformity should also be considered by surgeons. In our study, although pneumonia and delayed wound healing were observed as perioperative complications, none of the patients required surgical intervention to manage these complications. Internal fixation failure is a common complication of scoliosis surgery in SMA patients during long-term follow-up [8]. Notably, in our study, no mechanical complications occurred between the perioperative period and the last follow-up. We consider that the use of multi-rod constructs and S2-AI screws increased the stability of the internal fixation system and reduced the incidence of internal fixation failure.

This study had some limitations. This was a retrospective study with a relatively small sample size that focused on a specific patient group. However, given the rarity of SMA patients, this study comprises the largest sample size to date for assessing the outcomes of surgery for SMA patients with severe scoliosis and PO. We also provided a reference for patients with severe neuromuscular scoliosis. A conservatively treated group was not included, which would have facilitated further clarification of the natural history of SMA. Currently, no prospective longitudinal studies have compared the clinical outcomes of surgery and conservatively treated SMA scoliosis. Randomization of patients with SMA and severe scoliosis to a conservative treatment group is challenging, which is otherwise the standard treatment for such patients.

## Conclusion

Our findings showed that operative treatment significantly improved radiographic parameters and sitting function and maintained pulmonary function with no serious complications during the follow-up period of at least 2 years, suggesting that, although challenging, PSF with pelvic fixation can be considered safe and effective for SMA patients with severe spinal deformity (minimum major coronal curve Cobb angle > 100° and PO > 20°). Spinal surgery preserved overall pulmonary function, with 50% of patients showing improvements in FVC. The observed declines in some patients are likely due to the natural progression of spinal muscular atrophy rather than the impact of the surgical intervention. This study provides evidence and confidence in support of surgical treatment for patients with SMA and severe spinal deformity, improves patient counseling, and facilitates future research efforts.

## Data Availability

The datasets used or analysed during the current study are available from the corresponding author on reasonable request.

## Abbreviations

ANOVA: Analysis of variance
FVC: Forced vital capacity
IQRs: Interquartile ranges
MDSQ: Muscular dystrophy spine questionnaire
PO: Pelvic obliquity
PSF: Posterior spinal fusion
SD: Standard deviation
SMA: Spinal muscular atrophy
